# Supplementary material for: Engineering CRISPR immune systems conferring GLRaV-3 resistance in grapevine
Source: Hortic Res. 2022 Jan 28;9:uhab023. doi: 10.1093/hr/uhab023 (PMC8796251; doi:10.1093/hr/uhab023)
Supplement: Web_Material_uhab023 [file web_material_uhab023.zip › HORTRES-04369R2.docx]

**Engineering CRISPR immune systems conferring GLRaV-3 resistance in grapevine**

**Running Head: Engineering CRISPR systems conferring GLRaV-3 resistance**

Bolei Jiao^1†^, Xinyi Hao^1†^, Zhiming Liu^1^, Mingbo Liu^1^, Jingyi Wang^1^, Lin Liu^1^, Na Liu^1^, Rui Song^3^, Junxiang Zhang^3^, Yulin Fang^2^, Yan Xu^1*^

^1^State Key Laboratory of Crop Stress Biology in Arid Areas, College of Horticulture, Northwest A&F University, Yangling, Shaanxi, 712100, China

^2^College of Enology, Northwest A&F University, Yangling, Shaanxi, 712100, China

^3^Chinese Wine Industry Technology Institute, Zhongguancun Innovator Center, Yinchuan, Ningxia, 750000, China

^†^These authors contributed equally to this study.

^*^**Corresponding author:** Yan Xu

E-mail: [yan.xu@nwsuaf.edu.cn](mailto:yan.xu@nwsuaf.edu.cn)

Tel/fax +86-029-87082429

No. 3 Taicheng Road, College of Horticulture, Northwest A&F University, Yangling, Shaanxi, 712100, China

**E-mail addresses of other authors:**

Bolei Jiao, [guanjunberry@nwafu.edu.cn](mailto:guanjunberry@nwafu.edu.cn); Xinyi Hao, [xinyihao@nwsuaf.edu.cn](mailto:xinyihao@nwsuaf.edu.cn);

Zhiming Liu, [769858702@qq.com](mailto:769858702@qq.com); Mingbo Liu, lmb@nwafu.edu.cn;

Jingyi Wang, [3544972521@qq.com](mailto:3544972521@qq.com); Lin Liu, [1498393936@qq.com](mailto:1498393936@qq.com);

Na Liu, liuna0403@nwafu.edu.cn; Rui Song, 578071747@qq.com;

Junxiang Zhang, [zhangjunxiang@126.com](mailto:zhangjunxiang@126.com); Yulin Fang, fangyulin@nwsuaf.edu.cn

**Abstract:** Grapevine leafroll-associated virus 3 (GLRaV-3) is one of the causal agents of grapevine leafroll disease (GLD), which severely impacts grapevine production in most viticultural regions of the world. The development of virus-resistant plants is a desirable strategy for the efficient control of viral diseases. However, natural resistant resources have not been reported in the genus *Vitis,* and anti-GLRaV-3 research has been quite limited in grapevine. In this study, by expressing FnCas9 and LshCas13a, we established a highly effective transgenic construct screening system via an optimized *Agrobacterium*-mediated transient delivery system in grapevine plantlets. Our study indicated that CRISPR/FnCas9 and LshCas13a caused GLRaV-3 inhibition. Moreover, three vectors—pCR01-CP, pCR11-Hsp70h and pCR11-CP—exhibited the most robust inhibition efficiency compared to those targeting other sites and could be further engineered to generate GLRaV-3-resistant grapevine. In addition, the viral interference efficiency of FnCas9 was dependent on its RNA binding activity. The efficiency of virus inhibition was positively correlated with the level of Cas gene expression. Importantly, we demonstrated that LshCas13a had better interference efficiency against viruses than FnCas9. In summary, this study confirmed that these two RNA-targeting CRISPR mechanisms can confer immunity against viruses in grapevine, providing new avenues to control GLRaV-3 or other RNA viruses in fruit crops.

**Keywords:** GLRaV-3, CRISPR, FnCas9, LshCas13a, resistance, grapevine.

**Introduction**

Grapevine (*Vitis vinifera* L.) is widely cultivated around the world as a highly profitable crop^1^. However, virus-induced disease is one of the most serious biotic factors limiting the sustainable development of agricultural productivity^2-4^. Grapevine leafroll disease (GLD) has been recognized as a major threat to grapes across grape-growing regions of the world^5^. GLD causes significant yield losses (up to 30-68%), a drastic reduction in nutrients and anthocyanin biosynthesis in berries, and inhibition of leaf photosynthesis and plant growth^6-11^. The annual economic cost of viral infection is estimated to be up to $25,000 per hectare when no control measures are implemented^12^. Grapevine leafroll-associated virus-3 (GLRaV-3), a monopartite, positive-strand RNA virus, is considered the main etiological agent of GLD^5,13^.

Efforts to control GLRaV-3 have been attempted by using antiviral drugs^14,15^, virus-free plants^16^, vector management^17^ and conventional breeding. However, the risk of viral reinvasion, increasing tolerance to drugs, undesirable environmental disruption and rising costs still pose a serious threat to long-term disease management, since natural resources resistant to GLRaV-3 have not been identified in *Vitis* spp.^18,19^. Therefore, the development of virus-resistant plants is a desirable strategy for the efficient control of viral diseases. Conventional breeding is promising as an antiviral approach, but combining antiviral traits with other superior traits is difficult, time-consuming and costly. Although antiviral molecular research is quite limited in grapevine, various strategies to control plant viruses using transgenic approaches have been adopted in other species. For example, one of the major mechanisms involves RNA silencing, which has been reported to successfully confer resistance against viruses by introducing virus-derived genes and RNA sequences^20-22^. The disease resistance (R) gene was introduced into crop plants to enhance resistance to viral invasion^23,24^. Although these approaches are promising, many of their drawbacks necessitate innovative strategies for disease control.

The clustered regularly interspaced short palindromic repeats and associated proteins (CRISPR/Cas) systems had the ability to confer immunity against invasion by phages and conjugative plasmids in bacteria and archaea^25,26^. Based on the powerful ability of CRISPR/Cas systems to target DNA/RNA *in vivo*^27^, two strategies have been employed to obtain viral resistance, namely, direct targeting of viral genomic DNA/RNA^28-32^ and destruction of an essential host factor^33,34^. Nevertheless, it has been reported that the former strategy using the classic CRISPR/Cas9 system accelerated the progress of CRISPR-induced DNA virus evolution^35^, while the latter led to a risk of impaired plant growth^36^. Recent studies showed that certain variants of Cas proteins, namely, Cas9 from *Francisella novicida* (FnCas9) and Cas13a from *Leptotrichia shahii* (LshCas13a), function as RNA-guided RNA targeting effectors to bind to or cleave the viral RNA^37,38^, providing encouraging opportunities to combat plant RNA viruses^31,32,39-41^.

In this study, we sought to test the possibility of developing CRISPR-induced resistance against GLRaV-3 in grapevine. To this end, we optimized the *Agrobacterium*-mediated transient expression approach for *in vitro* plantlets. Next, CRISPR/FnCas9 and CRISPR/LshCas13a were introduced into grapevine through the optimized transient delivery system. The results demonstrated that interference against GLRaV-3 via these two CRISPR systems was significantly efficient in grapevine. The efficiency of inhibition against GLRaV-3 was positively correlated with the expression level of the FnCas9/LshCas13a gene. Importantly, crRNA-LshCas13a showed a higher interference efficiency than sgRNA-FnCas9. The three vectors described here could be used in further studies to generate virus-resistant plants because they exhibited high interference efficiency.

**Results**

**Establishing an optimum *Agrobacterium*-mediated transient transformation protocol *in planta***

In the present study, the β-glucuronidase (GUS) reporter gene, driven by an enhanced 35S promoter, was used to replace FnCas9 and LshCas13a in the pCR01 and pCR11 vectors, respectively (Fig. 1a). Subsequently, Agrobacterium GV3101 harboring these two vectors, which were named pCR01-GUS and pCR11-GUS, was used for the transient transformation assay through Agrobacterium-mediated vacuum infiltration in the *V. vinifera* cv. ‘Cabernet Sauvignon’.

To establish an effective *Agrobacterium*-mediated transient transformation method, the effects of the four most important factors, namely, *Agrobacterium* density, suspension pH, acetosyringone (AS) concentration and infection time, on the transformation were evaluated. The plantlets were submerged in an *Agrobacterium* bacterial suspension and vacuum infiltrated for 20 min, followed by quick release for 5 min, which was repeated three times (Fig. 1b). As shown in Fig. 2a, GUS staining was considered successful when uniform dark blue staining was found in the leaves of agroinfiltrated plantlets. In contrast, the unsuccessful case of transient transformation was unevenly stained with light blue spots. The transformation efficiency was calculated as the number of successfully stained plants/total number of plants. In addition, mock plantlets infected with empty *Agrobacterium* suspension are shown in Supplementary Figure 1. The highest transformation efficiency (93.33% for both pCR01-GUS and pCR11-GUS) was obtained at an optical density at 600 nm

**Fig. 1 *Agrobacterium*-mediated transient delivery assay in grapevine plantlets. a** Schematic illustration of the plasmid profiles of pCR01-GUS and pCR11-GUS. **b** Transient expression protocol with vacuum infiltration in grapevine plantlets. Six-week-old plantlets were submerged in an *Agrobacterium* GV3101 strain bacterial suspension harboring the test vector resuspended at a final OD_600_ of 1.0 and were vacuum infiltrated in a Nucerite desiccator for 60 min, followed by a quick pressure release.


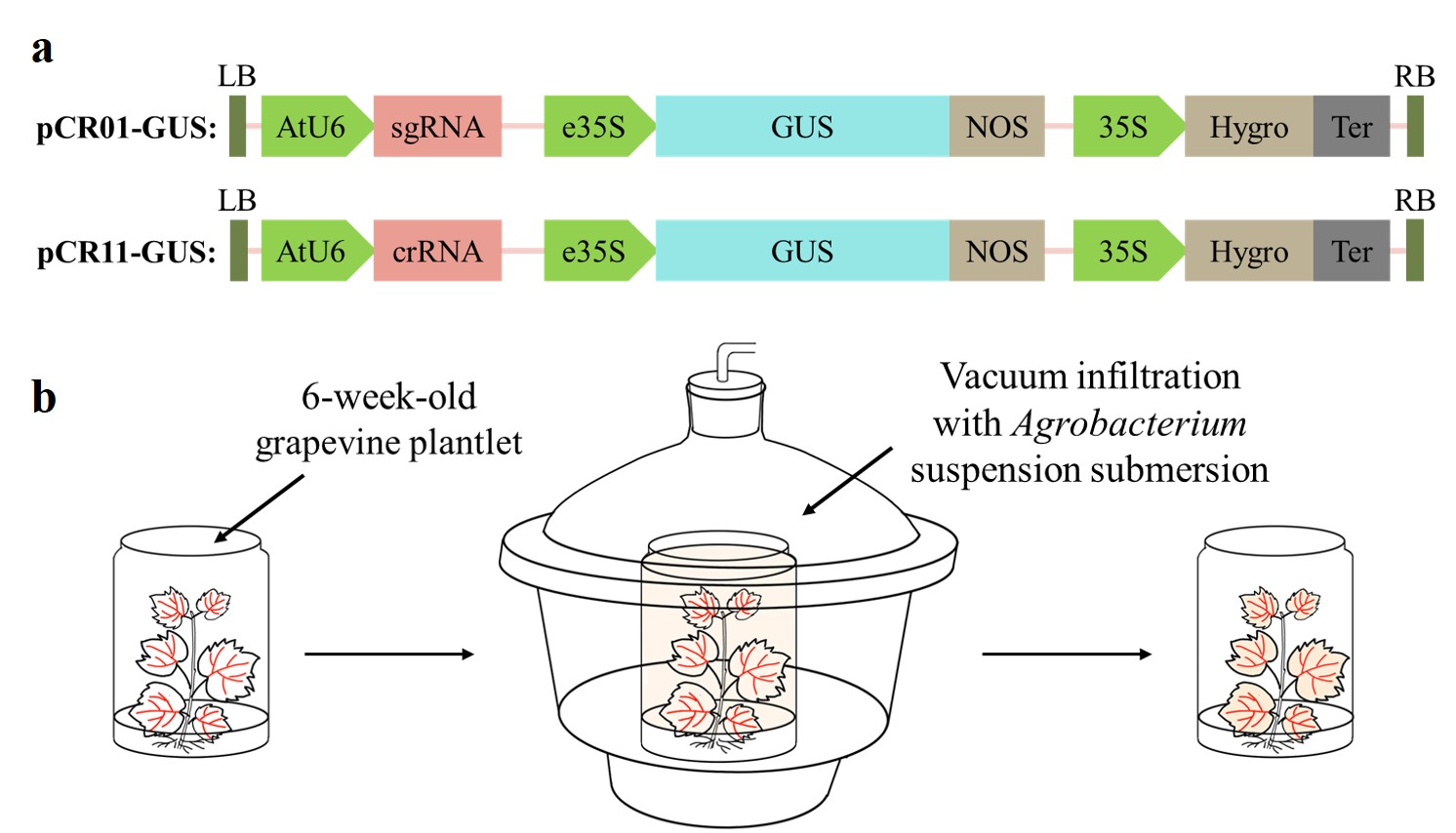


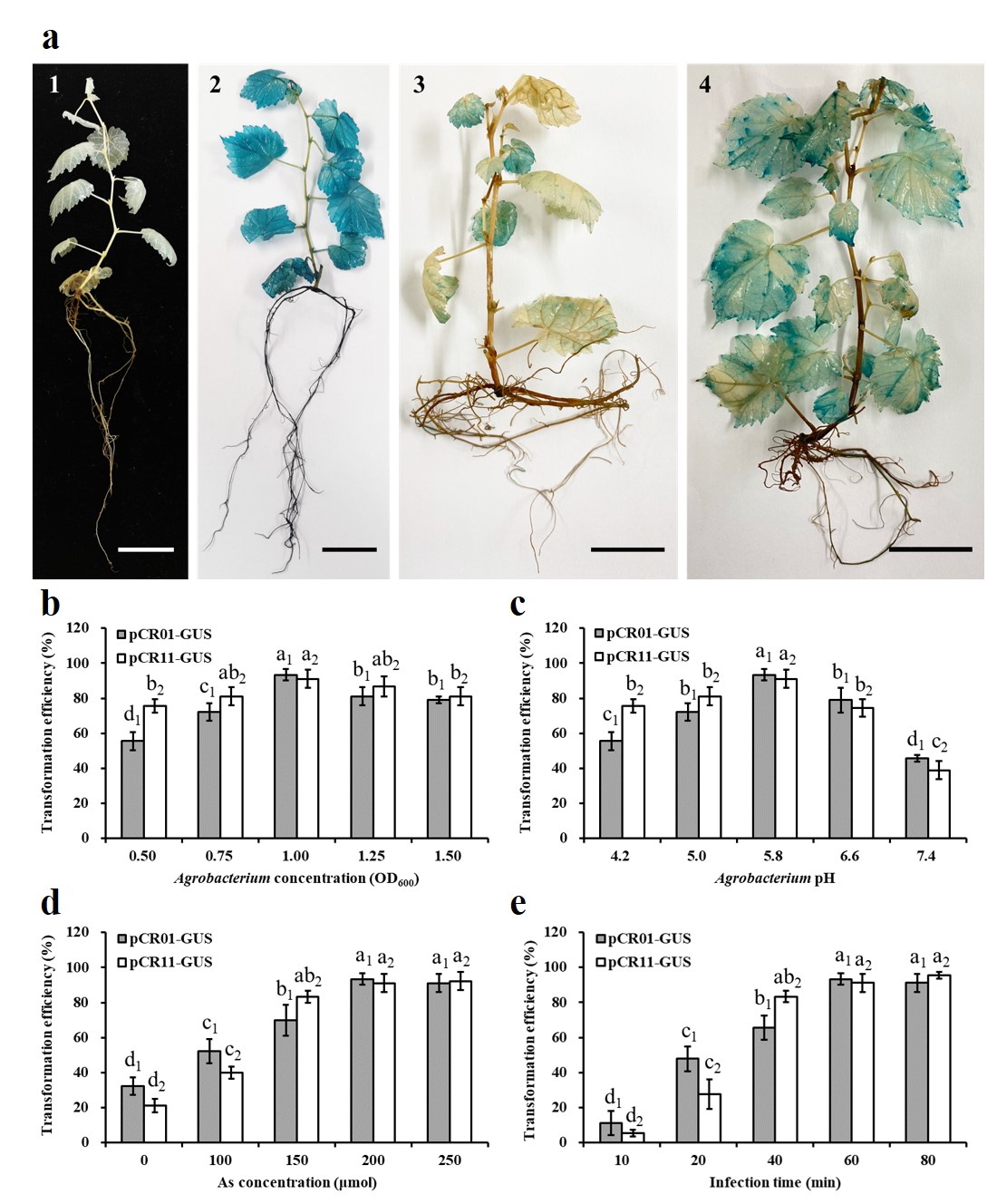


(OD_600_) of 1.00. The transformation efficiency decreased at higher or lower suspension concentrations (Fig. 2b). The highest transformation efficiency was obtained with both vectors at a suspension pH value of 5.8. Compared with the alkaline bacterial suspensions (pH=7.4), acidic bacterial suspensions (pH=5.0 and 4.2) significantly enhanced *Agrobacterium* infection (Fig. 2c). The number of positive GUS plantlets increased significantly with increase in AS level, and the transformation efficiency peaked and plateaued at an AS concentration of 200 µM (93.33% of pCR01-GUS and pCR11-GUS) (Fig. 2d). An infection time of 60 min resulted in the highest efficiency of 93.33% (pCR01-GUS and pCR11-GUS) (Fig. 2e). A shorter infection time reduced the transformation efficiency, and longer infection could damage the plantlets.

**Fig. 2 GUS staining observations and analysis of transient expression results. a** Histochemical assay of GUS expression observed in ‘Cabernet Sauvignon’ plantlets. **a1** shows the control plantlet. **a2** shows the plantlet that was stained successfully. **a3, a4** show the plantlets that were not completely stained. Bar = 1 cm. **b** Effect of *Agrobacterium* concentration on transient transformation (OD_600_ values of 0.5, 0.75, 1.0, 1.25, and 1.5). **c** Effect of Agrobacterium pH on transient transformation (4.2, 5.0, 5.8, 6.6, and 7.4). **d** Effect of AS concentration on transient transformation (0, 100, 150, 200, and 250 μM). **e** Effect of infection time on transient transformation (10, 20, 40, 60, and 80 min). Each replicate comprised 10 *in vitro* plantlets, and every treatment had three replicates. The transformation efficiency was calculated as number of successfully stained plants/total number of plants. The data are presented as the means ± SEs, and significant differences were analyzed with SPSS using one-way ANOVA with the Tukey test; different letters indicate a significant difference at *P* < 0.05.

For pCR01-GUS and pCR11-GUS, at 5 days postinfection (dpi), the best performance occurred at an OD_600_ of 1.0, pH value of 5.8, AS concentration of 200 μM, infection time of 1 h, and a vacuum pressure of -0.8 MPa. The efficiency reached up to 96.67%. Every treatment consisted of three replicates, and 10 plantlet samples were used in each replicate. In this study, the transformation efficiency of the two vectors showed a similar tendency. We also engineered this assay in *V. vinifera* cv. ‘Kyoho’ (Supplementary Figure 2). These results demonstrated that we established an efficient *Agrobacterium*-mediated transient transformation method for grapevine plantlets.

**Engineering CRISPR/FnCas9 and LshCas13a machinery for *in planta* expression**

The vectors pCR01 and pCR-11 comprised an sgRNA or crRNA driven by an AtU6 promoter and a Cas gene driven by an enhanced 35S promoter (Fig. 3a). To target the GLRaV-3 virus, the complete genome sequence of GLRaV-3-Sau was obtained and deposited in NCBI GenBank (accession number MK988555). The genome of GLRaV-3 contains 5 conserved ORFs, ORF-4-8, which are a conserved hallmark of the family *Closteroviridae*^13,42^. We selected ten target sites within these ORFs; both of the CRISPR systems contained five targets, namely, a 5 kDa protein (p5), heat stimulated protein 70 homolog (Hsp70h), heat stimulated protein 90 homolog (Hsp90h), coat protein (CP) and minor coat protein (CPm) sequences (Fig. 3b). Next, we synthesized and inserted sgRNA into pCR01 and crRNA into pCR11.

To confirm the expression of FnCas9 and LshCas13a in transient assays in grapevine, we employed pCR01 and pCR11 using our optimized transformation system. FnCas9 was fused with a 3×FLAG tag in pCR01, and LshCas13a was fused with a 3×HA tag in pCR11. Western blotting results showed that Cas proteins were successfully expressed in plantlets, and proteins of the expected size were detected (163 kDa for FnCas9 and 156 kDa for LshCas13a) (Fig. 3c). To investigate the expression of Cas genes in different periods, RT–qPCR was carried out, and the results demonstrated that the relative expression of Cas genes reached a peak at 5 dpi (Fig. 3d). These results indicated that FnCas9 and LshCas13a were efficiently expressed in plant leaves.

**Fig. 3 Expression of the pCR01 and pCR11 vectors.** **a** Schematic diagrams of the pCR01 and pCR11 vectors used in this study. **b** The conserved sequence region of GLRaV-3 and corresponding target ID. P5: 5 kDa protein; Hsp70h: heat shock protein 70 homolog; Hsp90h: heat shock protein 90 homolog; CP: coat protein; CPm: minor coat protein. We chose A-E as targets to design sgRNA/crRNA. **c** Western blotting validation of FnCas9/LshCas13a protein expression. **d** Relative expression level of Cas genes at different time points after an *Agrobacterium*-mediated transient transformation assay.


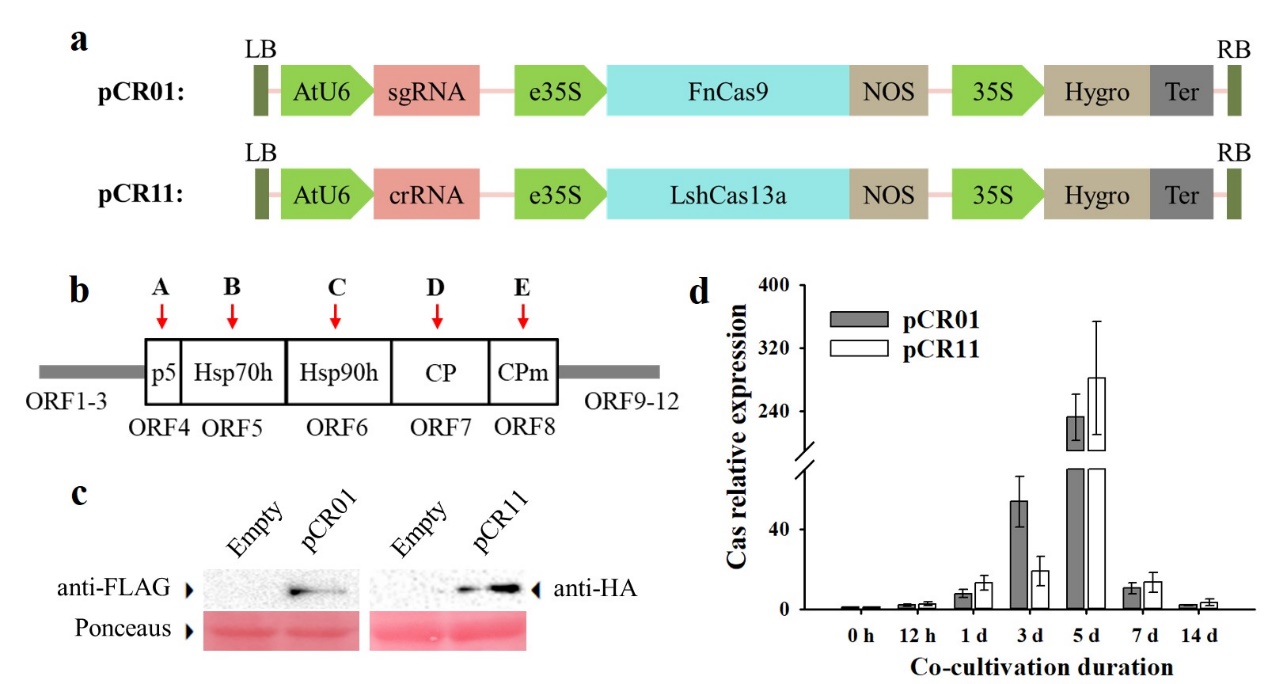


**CRISPR/FnCas9 interferes with GLRaV-3 in grapevine**

To investigate the inhibitory effect of sgRNA-FnCas9 on GLRaV-3, optimized transient expression assays were carried out to transfer agrobacterial suspensions containing the experimental vector or control vector into 6-week-old GLRaV-3-infected *in vitro* stock shoots. Additionally, the virus concentration in the different virus-infected plantlets was at the same level before infiltration. Then, plantlets were cultured in darkness at 24 ℃ for 12 h. Next, after washing softly, shoots (1.5 cm in length) with one fully opened leaf were excised from plantlets and cultured on BM medium containing 4% PEG. After 2 weeks, the typical GLRaV-3 symptoms described above were found on virus-infected control shoots (VI+Mock) and virus-infected plants with nonspecific sgRNA-FnCas9 constructs (VI+pCR01-ns). However, only light reddish leaf symptoms were observed in the experimental groups compared with either VI+Mock or VI+pCR01-ns (Fig. 4a). In shoots infiltrated with sgRNA-FnCas9 constructs, the expression of sgRNAs was successfully detected by an RT–PCR assay (Fig. 4b), and FnCas9 proteins were also confirmed by western blotting (Fig. 4c). Consistent with the symptoms, quantification of viral RNA by RT–qPCR indicated a significant reduction in all five experimental groups except for pCR01-1C compared with VI+Mock and VI+pCR01-ns (Table 1). Among these groups, pCR01-1D exhibited a substantial reduction in viral accumulation, up to 86%, while pCR01-1C exhibited a mild reduction, only 22%. Other constructs reduced viral RNA by 40-70%.

**Table 1.** Correlation between the FnCas9 expression level and GLRaV-3 accumulation

|  | Healthy | Mock | pCR01-ns | pCR01-1A | pCR01-1B | pCR01-1C | pCR01-1D | pCR01-1E |
| --- | --- | --- | --- | --- | --- | --- | --- | --- |
| FnCas9 | 0.00±0.00e | 0.00±0.00e | 1.01±0.14bc | 0.77±0.10cd | 0.59±0.04d | 1.18±0.18b | 1.62±0.23a | 1.35±0.17ab |
| GLRaV-3 | 0.00±0.00e | 1.06±0.09ab | 1.08±0.11a | 0.46±0.08cd | 0.60±0.08c | 0.84±0.02b | 0.15±0.09ef | 0.30±0.10de |
| FnCas9 ×GLRaV-3 correlation coefficient | | | | -0.499^*^ | Sig. | 0.025 | N | 20 |

Different letters and * are significantly different at *P* < 0.05

To investigate the correlation between relative FnCas9 expression and viral accumulation, RT–qPCR of FnCas9 expression was also carried out, and a correlation analysis of these two variables was performed (Table. 1). Relative FnCas9 expression was negatively correlated with viral accumulation at the *P* < 0.05 level. The Pearson correlation coefficient was -0.499.

**FnCas9 could bind GLRaV-3 RNA via sgRNA guidance**

To investigate whether FnCas9 could directly bind GLRaV-3 genomic RNA by specific sgRNA guidance, the most efficient vector pCR01-1D, together with the control vector pCR01-ns, was selected to perform the RNA coimmunoprecipitation (RIP) assay. First, total protein was extracted from leaf samples using protein extraction buffer, which was modified according to Pang *et al*.^43^. Then, FnCas9 was immunoprecipitated using FLAG antibody, and the RNA associated with FnCas9 was purified. Next, GLRaV-3 and sgRNA were detected by RT–PCR. Western blotting analysis showed that FnCas9 proteins were successfully expressed and immunoprecipitated, and sgRNAs were also precipitated by binding to FnCas9. Finally, GLRaV-3 CP RNA was present only in virus-infected plantlets with pCR01-1D (Fig. 4d), while it was not detectable in other samples inoculated with nonspecific sgRNA. These analyses indicated that FnCas9 could bind to viral RNA via specific sgRNA guidance in plants.

**CRISPR/LshCas13a inhibits viral accumulation**

To assess the interference activity of crRNA-LshCas13a against GLRaV-3, optimized *Agrobacterium*-mediated transient expression was used *in planta,* and the procedure was performed as described for FnCas9-mediated inhibition against virus. At

14 dpi, none of the leaves from healthy *in vitro* plantlets displayed any reddish coloration (Fig. 4e). While the virus-infected control (VI+Mock) and pCR11-ns control (VI+pCR11-ns) exhibited severe reddish-purple coloration, very limited typical GLRaV-3 symptoms were observed in the five experimental groups. RT–PCR results indicated that crRNAs were detectable in plantlets infiltrated with pCR11-crRNA (Fig. 4f). Western blotting analysis showed that LshCas13a proteins were also expressed in these plantlets, and the correct proteins were detected (Fig. 4g). Quantification of viral accumulation by RT–qPCR demonstrated that all crRNA-LshCas13a constructs significantly reduced viral replication by 60-90% compared with VI-Mock and pCR11-ns. The best interference against the virus was observed with crRNA targeting 2B and 2D. These data confirmed that LshCas13a guided by specific crRNA indeed suppressed viral accumulation.


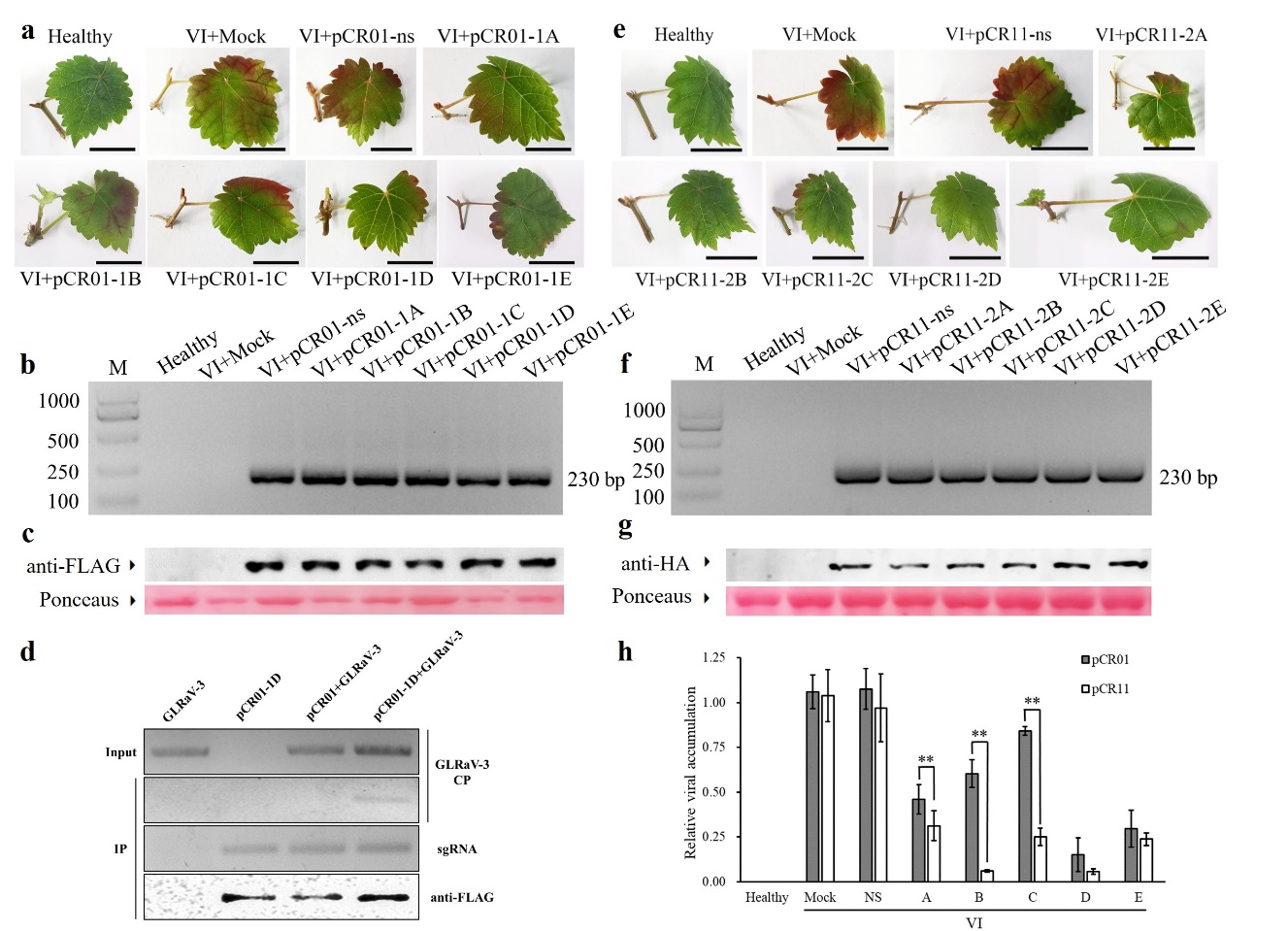


**Fig. 4 CRISPR-mediated resistance against GLRaV-3 in grapevine. a** Symptoms of 14-day-old GLRaV-3-infected (VI) basal plantlets transiently expressing pCR01-sgRNAs and control vectors. Scale bars = 1 cm. **b** sgRNA detection and **c** FnCas9 expression in VI shoots at 5 dpi. **d** FnCas9 targets GLRaV-3 RNA by direct binding. At 5 dpi, lysates from shoot leaves were immunoprecipitated with anti-FLAG antibody. The coprecipitating viral RNA and sgRNA were purified and analyzed by RT–PCR. Western blotting was performed to confirm that the FnCas9 proteins were successfully expressed and immunoprecipitated. **e** Disease symptoms of 14-day-old VI basal plantlets transiently expressing pCR11-crRNAs and control vectors. Scale bars = 1 cm. **f, g** Expression of crRNA-LshCas13a was confirmed. **h** Comparative analysis of the efficiency of virus inhibition between CRISPR/FnCas9 and CRISPR/LshCas13a. Significance analysis was adopted using the independent samples t-test. ** indicates significant differences at *P*＜0.01.

Correlation analysis of Cas gene expression levels and viral accumulation was conducted, and the results showed that the expression level of LshCas13a was significantly negatively correlated with viral accumulation at the *P* < 0.01 level (Table 2). The Pearson correlation coefficient was -0.572.

**Table 2.** Correlation between the LshCas13a expression level and viral accumulation

|  | Healthy | Mock | pCR11-ns | pCR11-2A | pCR11-2B | pCR11-2C | pCR11-2D | pCR11-2E |
| --- | --- | --- | --- | --- | --- | --- | --- | --- |
| LshCas13a | 0.00±0.00d | 0.00±0.00d | 1.05±0.21b | 0.51±0.16c | 0.98±0.26b | 1.19±0.21b | 1.62±0.49a | 1.03±0.16b |
| GLRaV-3 | 0.00±0.00d | 1.04±0.15a | 0.97±0.19a | 0.31±0.08b | 0.06±0.01c | 0.25±0.05b | 0.06±0.02c | 0.24±0.04b |
| LshCas13a ×GLRaV-3 correlation coefficient | | | | -0.572^***^ | Sig. | 0.000 | N | 20 |

Different letters are significantly different at *P* < 0.05, *** is significantly different at *P* < 0.001

**LshCas13a exhibited a better virus inhibition efficiency than FnCas9**

Finally, we compared the efficiency of viral disruption of FnCas9 with that of LshCas13a by an independent sample t-test (Fig. 4h). The results showed that all sequence-specific crRNA-LshCas13a constructs had stronger interference with viral replication than sgRNA-FnCas9 targeting the same targets. In addition, these three targets, namely, 2A, 2B and 2C, of crRNA-LshCas13a showed a significant reduction in viral accumulation compared to sgRNA-FnCas9 targeting 1A, 1B and 1C, whereas no significant difference was observed between the two CRISPR systems in the D and E targets. Therefore, these results indicated that LshCas13a could suppress viral replication and accumulation better than FnCas9 in grape.

**Discussion**

GLRaV-3 causes severely damaging disease in grapevine^44^. However, to date, studies on establishing resistance against GLRaV-3 in grapevine have been quite limited. This is the first report of specific targeting of the GLRaV-3 RNA genome for attenuation of viral infection. In this study, the antiviral CRISPR system was successfully employed to impart resistance against GLRaV-3 in transient assays.

First, we optimized the *Agrobacterium*-mediated transient transfection method in 6-week-old plants grown *in vitro*. Our study demonstrated that the highest transformation efficiency of ‘Cabernet Sauvignon’ grown *in vitro* occurred with an OD_600_ of 1.0, infection time of 60 min, AS concentration of 200 μM and pH value of 5.8. Compared with previous studies, our methods could achieve high transformation efficiency without mechanical damage^45,46^. In previous studies, to promote agroinfection, the leaves were damaged by enlarging injured areas, which may affect plant growth activity^47-50^ and may even have undesirable adverse effects on the results^51^.

By designing specific guide RNAs (sgRNA/crRNA) against conserved coding regions of GLRaV-3, the CRISPR/FnCas9 and CRISPR/LshCas13a systems were engineered to effectively improve viral inhibition efficiency in grapevine. A recent study demonstrated that a 40-80% reduction in cucumber mosaic virus (CMV) accumulation was caused by transiently delivering sgRNA-FnCas9 in *Nicotiana benthamiana* compared to the control^31^. The CRISPR/FnCas9 system engineered in our study resulted in a reduction in viral accumulation of up to 86% with pCR01-Hsp90h (Table 1), indicating better interference against RNA viruses in grapevine than in model plants. RNA targeting of LshCas13a also offers an alternative anti-GLRaV-3 strategy^37^. All CRISPR/LshCas13a vectors showed similar efficiency of viral interference in *N. benthamiana*, potato and rice^41,52^. However, our results confirmed that crRNA targeting Hsp70h and CP produced much higher LshCas13a-mediated virus inhibition efficiency than that of other targets (Table 2). Likewise, Aman *et al*.^32^ reported a significant difference in viral inhibition between the targets HC-Pro and GFP. Thus, the vectors that we selected could be further used to establish resistance against GLRaV-3 in stable transgenic lines.

A previous study indicated that the expression level of LshCas13a was positively correlated with the inhibition efficiency against *potato virus Y* (PVY)^52^, which was consistent with our findings. We further found that a positive correlation was also observed in the CRISPR/FnCas9-mediated antiviral experiment, although the correlation was moderate for FnCas9 and weak for LshCas13a (Tables 1, 2). In addition, using the RIP assay, we confirmed that FnCas9 could bind viral RNA via sgRNA guidance (Fig. 4d). The RNA binding activity of FnCas9, instead of cleavage activity, could block viral replication, which has also been reported in mammalian cell lines and other plants^31,39^. Interestingly, FnCas9 also showed highly specific binding to DNA targets that was strictly dependent on sgRNA matching with targets^53^. Given the difference in sequences between plants and viruses, we designed an appropriate specific sgRNA, resulting in FnCas9 binding to viral genomic RNA but not plant DNA. Thus, the adverse effects on plant growth could be circumvented by the low off-target binding affinity. Furthermore, in contrast to DNA targeting by Cas9, which relies on nuclear localization, the cytosolic localization of FnCas9 would potentially limit off-target effects on the host DNA^39^. The CRISPR/LshCas13a system was engineered to mediate the specific knockdown of RNA transcripts. This property would limit off-target effects in plant cells^37^.

By direct comparison of the viral accumulation levels of two CRISPR systems targeting the same coding region, we further found that the virus interference efficiency of LshCas13a was much higher than that of FnCas9 (Fig. 4h). Our results indicated that LshCas13a with RNase activity was an excellent candidate that could be applied to generate virus-resistant plants compared to FnCas9, which was dependent on the binding ability instead of cleavage activity. To date, many other Cas13 orthologs have been discovered and engineered to combat mammalian virus^54,55^, such as Cas13a from *Leptotrichia wadei* (LwaCas13a), Cas13b from *Prevotella* sp*. P5-125* (PspCas13b) and Cas13d from *Ruminococcus flavefaciens* XPD3002 (CasRx)^56-58^. Among these Cas13 subtypes, CasRx exhibited the most robust virus interference activity^59^. Such studies provided more options to control plant viruses based on CRISPR-mediated interference strategies. In addition, no reports thus far have shown that RNA viruses have evolved escape machinery against RNA-targeting CRISPR/Cas systems.

In conclusion, we generated resistance against GLRaV-3 by delivering sgRNA-FnCas9 and crRNA-LshCas13a into grapevine plantlets via transient expression. The three vectors that we selected have the potential to further establish viral resistance in grapevine via robust viral interference. In addition, CRISPR/LshCas13a, by harnessing RNase activity, showed better viral interference than CRISPR/FnCas9, which exhibited dependence on RNA binding, not cleavage ability. These two CRISPR-mediated antiviral strategies may be of value for establishing virus-resistant grapevine and other fruit crops.

**Materials and methods**

**Plant materials**

The red wine grapevine cultivar ‘Cabernet Sauvignon’ (*V. vinifera*) was used in this study. The sequence of the viral strain GLRaV-3-Sau was obtained and deposited in GenBank (accession number MK988555). Healthy and GLRaV-3-infected *in vitro* plantlets were established as described in our previous studies^61,62^, and all the experimental GLRaV-3-infected plantlets had similarly high viral concentrations^63^. We also established *V. vinifera* cv. ‘Kyoho’ *in vitro* stock shoots. All *in vitro* plantlets were cultured on basic medium (BM) containing half-strength Murashige and Skoog (1962) (MS) medium supplemented with 30 g/L sucrose and 7 g/L agar. The pH of the medium was adjusted to 5.8 before autoclaving at 121 °C for 20 min. Subculture was performed once every 5 weeks. The cultures were maintained at a constant temperature of 24 ± 2 °C under a 16/8 photoperiod. “Cabernet Sauvignon” was used to optimize the transient expression system for CRISPR-mediated viral inhibition. “Kyoho” was only used to test the optimized protocol.

**Target selection and vector construction**

The GUS gene was amplified by PCR from the pBI121 vector with the primers 01-GUS-F/01-GUS-R and 11-GUS-F/11-GUS-R (Supplementary Table S1). Then, the PCR product was inserted into pCR01 and pCR11, which had been digested using NcoI and BamHI, to produce the vectors pCR01-GUS and pCR11-GUS, respectively.

All the targets were selected manually in viral conserved ORFs (Fig. 3b). For FnCas9, sgRNAs were designed without consideration of the protospacer adjacent motif (PAM) types^31^. However, for LshCas13a, the design of crRNAs required the protospacer flanking sequence (PFS) of A, U, or C adjacent to the targeted region^32^. All the sgRNAs/crRNAs were 100% complementary to the target sequences. To avoid the potential effect of off-targets, we searched similar sequences of target sequences from the genome of *V. vinifera* (ID:401, NCBI) using the Basic Local Alignment Search Tool (<https://blast.ncbi.nlm.nih.gov/Blast.cgi>), and the identity was less than 68%. The variant vectors of pCR01-sgRNA and pCR11-crRNA were constructed as described previously^31,41^. Briefly, based on the conserved sequence ORF4-8 of GLRaV-3-Sau, sgRNAs and crRNAs were designed and constructed as primer dimers (Supplementary Table S2, S3), and the synthesized sgRNA oligos were ligated with pCR01 digested using BsaI. The synthesized crRNA oligos were ligated with pCR11 digested using BsaI. Finally, all the vectors were authenticated by sequencing and were separately transformed into *Agrobacterium tumefaciens* strain GV3101 via the freeze-thaw method^64^.

***Agrobacterium*-mediated transient assay *in planta***

*Agrobacterium* was cultured overnight in liquid LB medium containing 50 mg/L kanamycin (Kan), 50 mg/L gentamicin (Gent) and 50 mg/L rifampicin (Rif). Then, the *Agrobacterium* cells were collected by centrifugation and resuspended in suspension solution containing 10 mM MES and 10 mM MgCl_2_. In this study, four experiments were conducted to test the effects of *Agrobacterium* density, suspension pH, AS concentration and infection time on transformation efficiency in “Cabernet Sauvignon.”. I, Cells were suspended in the above suspension solution with 200 µM AS to an OD_600_ value of 0.50, 0.75, 1.00, 1.25 or 1.50 prior to adjusting the pH to 5.8. II, Cells were suspended in the above suspension solution in the presence of 200 µM AS to an OD_600_ of 1.00 and pH values of 4.2, 5.0, 5.8, 6.6, or 7.4. III, Cells were diluted and adjusted to an OD_600_ of 1.0, and then 0, 100, 150, 200 or 250 µM AS was added. Plantlets were submerged in suspension solutions I to III and then vacuum infiltrated with -0.8 MPa for 60 min; plantlets in group IV were infected with suspension solution supplemented with 200 µM AS at an OD_600_ of 1.0 and pH 5.8 for 10, 20, 40, 60, or 80 min. Vacuum infiltration was paused every 20 min with a quick pressure release.

In the CRISPR-mediated antiviral assays, after performing the optimized transient expression procedure, the plantlets were cultured in darkness for 12 h. Subsequently, these plantlets were rinsed three times with sterile distilled water containing antibiotics [300 mg/L cefotaxime (Cef) and 200 mg/L carbenicillin (Carb)]. Next, shoots (1.5 cm in length) with one fully opened asymptomatic leaf were excised from the plantlets and cultured on BM containing 4% PEG, which could promote the typical reddish-purple coloration on the leaf blades of virus-infected grapevine^63^, and maintained under a 16-h light/8-h dark photoperiod.

**GUS staining and observation**

GUS staining was performed according to Baltes *et al*.^65^. In brief, five days after infiltration, plantlets were immersed in GUS staining solution [10 mM EDTA, 0.5% X-Gluc, 1 mM ferricyanide, 1 mM ferrocyanide, 0.1% Triton X-100, 10 mM phosphate buffer (pH 7.0)] and incubated at 37 °C for ~24 h. Finally, chlorophyll was removed by submerging stained plantlets in 70% ethanol at 37 °C for 1 week. The staining was considered unsuccessful if any leaf or any part of the leaf was not stained.

**RNA extraction and one-step RT–qPCR**

To test the expression of Cas genes, RNA was extracted from basal leaves of plantlets. In the CRISPR-mediated antiviral assay, RNA was extracted from the leaves of shoots at 14 dpi. RNA extraction was conducted using an Omega Plant RNA Kit (Omega, Norcross, USA) according to the manufacturer's instructions. Then, 1 μg of RNA was used to synthesize cDNA using reagent kits (RR047A, Takara, Japan) as described previously^63^. RT–qPCR was performed with SYBR Premix Ex Taq TM II (Takara, Japan) using an Applied Biosystems QuantSudio^TM^ 6&7 instrument (Thermo Fisher Scientific, USA) and QuantStudio^TM^ Real-Time PCR System (Thermo Fisher Scientific, USA) according to the manufacturer’s protocols. The 18S ribosomal RNA of grapevine was used as an internal reference gene (qVvActin-F/qVvActin-R: GTGACGGAGAATTAGGGTTCGA/CTGCCTTCCTTGGATGTGGTA)^66^. The oligonucleotide primers used for RT–qPCR are listed in Supplementary Table S1.

**Protein extraction and western blotting**

To validate the expression of Cas proteins, agroinfiltrated leaves were picked at 5 dpi, and protein extraction and western blotting were performed as described by Chen *et al*.^67^.

**Immunoprecipitation**

Protein was extracted as described by Pang *et al*.^43^ with modification. Briefly, 1 g of homogenized powder of leaf samples was resuspended in 3 mL of protein extraction buffer [0.05 M phosphate buffer (pH 7.0), 1 M DTT, 2% PVP, 50 μL of complete protease inhibitor cocktail (one pill of protease inhibitor was dissolved in 1 mL of ddH_2_O) (Roche Diagnostics GmbH, Germany), and 10 U/mL RNase inhibitor (Thermo Fisher Scientific Inc., USA)]. Then, vortexing and centrifugation were conducted prior to collection of the supernatant.

RT–PCR was performed to detect GLRaV-3 RNA with the primers GLRaV-3-F/GLRaV-3-R. Protein samples and FLAG-specific monoclonal antibodies were incubated with RNase inhibitor (Thermo Fisher Scientific Inc, USA) overnight at 4 °C. Next, the immune complexes were incubated with Protein A+G Sepharose (Seven Sea Pharmatech Co., Ltd, China) for 4–6 h at 4 °C. Then, the supernatant was discarded after centrifugation. Subsequently, the precipitated fraction was washed with immunoprecipitation buffer [0.05 M phosphate buffer (pH 7.0), 10% glycerol, 1 M DTT, 2% PVP, 0.2% Triton X-100, 50 μL of complete protease inhibitor cocktail, and 10 U/mL RNase inhibitor]. The immunoprecipitated FnCas9 proteins were verified by 10% SDS–PAGE. RT–PCR was carried out to detect GLRaV-3 CP RNA and sgRNA from precipitates with the primers GLRaV-3-CP-F/GLRaV-3-CP-R and the primers sgRNA-F/sgRNA-R, respectively.

**Data analysis**

For transient expression experiments, every treatment consisted of three replicates, and 10 plantlet samples were used in each replicate. The data are presented as the means ± SEs, and significant differences were analyzed with SPSS using one-way ANOVA with the Tukey test; different letters indicate significant differences at *P* < 0.05. The transformation efficiency was calculated as the number of successfully stained plants/total number of plants. Three replicates were included in every treatment, and each replicate consisted of three technical replicates for RT–qPCR assays. Significant differences among mean values were analyzed by one-way ANOVA with Tukey’s test, and different letters with the same vector are significantly different at *P* < 0.05. Pearson correlation analysis was conducted to examine whether the expression level of FnCas9/LshCas13a was correlated with viral accumulation. The significance of the differences between two variables was assessed by an independent-samples t-test.

**Data availability**

The data that support the findings of this study are available from the corresponding author upon reasonable request.

**Acknowledgments**

We thank Dr. Guohui Zhou and Dr. Tong Zhang for sharing their vector plasmids. We also thank Dr. Xuequn Pang for helping with the RIP assay and Dr. Qiaochun Wang and Dr. Minrui Wang for kindly helping with manuscript revision. This research was funded by the National Key Research and Development Program of China (Item no. 2019YFD1002500), Ningxia Hui Autonomous Region Key Research and Development Program (2021BEF02015), Ningxia Hui Autonomous Region Flexible Introduction Team Project (2020RXTDLX08), and Shaanxi Province Agriculture Research Systems for the Grape Industry.

**Conflict of interest**

Y.X. and B.J. have filed a patent application in China (priority filing with serial number 202011040617.8).

**Authors’ contributions**

Y.X., B.J. and X.H. conceived the research; B.J. and X.H. designed the experiments; B.J., Z.L., M.L., J.W., L.L. and N.L. performed the experiments; B.J., R.S., J.Z. and Y.F. analyzed the results; B.J. and Y.X. wrote the manuscript; and Y.X. revised the manuscript. All authors read and approved the final manuscript.

**References**

1. Bacilieri, R. *et al.* Genetic structure in cultivated grapevines is linked to geography and human selection. *BMC Plant Biol.* **13**, 25 (2013).

2. Tilman, D., Balzer, C., Hill, J. & Befort, B. L. Global food demand and the sustainable intensification of agriculture. *Proc. Natl. Acad. Sci. U S A* **108**, 20260-20264 (2011).

3. Whitham, S.A., Yang, C. & Goodin, M. M. Global impact: Elucidating plant responses to viral infection. *Mol. Plant-Microbe Interact.* **19**, 1207-1215 (2006).

4. Martelli, G. P. Directory of virus and virus-like diseases of the grapevine and their agents. *J. Plant Pathol.* **96**, 1-136 (2014).

5. Naidu, R. A., Maree, H. J. & Burger, J. T. Grapevine leafroll disease and associated viruses: A unique pathosystem. *Annu. Rev. Phytopathol.* **53**, 613-634 (2015).

6. Sampol, B., Bota, J., Riera, D., Medrano, H. & Flexas, J. Analysis of the virus-induced inhibition of photosynthesis in malmsey grapevines. *New Phytol.* **160**, 403-412 (2003).

7. Gutha, L. R., Casassa, L. F., Harbertson, J. F. & Naidu, R. A. Modulation of flavonoid biosynthetic pathway genes and anthocyanins due to virus infection in grapevine (*Vitis vinifera* L.) leaves. *BMC Plant Biol.* **10**, 187 (2010).

8. Vega, A., Gutierrez, R. A., Pena-Neira, A., Cramer, G. R. & Arce-Johnson, P. Compatible GLRaV-3 viral infections affect berry ripening decreasing sugar accumulation and anthocyanin biosynthesis in *Vitis vinifera*. *Plant Mol. Biol.* **77**, 261-274 (2011).

9. Moutinho-Pereira, J. *et al.* Impacts of leafroll-associated viruses (GLRaV-1 and -3) on the physiology of the Portuguese grapevine cultivar ‘Touriga Nacional’ growing under field conditions. *Ann. Appl. Biol.* **160**, 237-249 (2012).

10. Halldorson, M. M. & Keller, M. Grapevine leafroll disease alters leaf physiology but has little effect on plant cold hardiness. *Planta* **248**, 1201-1211 (2018).

11. Habili, N. & Nutter, F. W. Temporal and spatial analysis of grapevine leafroll-associated virus 3 in Pinot Noir grapevines in Australia. *Plant Dis.* **81**, 625-628 (1997).

12. Atallah, S. S., Gomez, M. I., Fuchs, M. F. & Martinson, T. E. Economic impact of grapevine leafroll disease on *Vitis vinifera* cv. Cabernet franc in finger lakes vineyards of New York. *Am. J. Enol. Viticult.* **63**, 73-79 (2011).

13. Maree, H. J. *et al.* Grapevine leafroll-associated virus 3. *Front. Microbiol.* **4**, 82 (2013).

14. Panattoni, A., D'Anna, F. & Triolo, E. Antiviral activity of tiazofurin and mycophenolic acid against Grapevine leafroll-associated virus 3 in *Vitis vinifera* explants. *Antiviral Res.* **73**, 206-211 (2007).

15. Wang, X. Y. *et al.* Crude garlic extract significantly inhibits replication of grapevine viruses. *Plant Pathol.* **69**, 149-158 (2019).

16. Wang, Q. & Valkonen, J. P. Cryotherapy of shoot tips: Novel pathogen eradication method. *Trends Plant Sci.* **14**, 119-122 (2009).

17. Tsai, C. W. *et al.* Transmission of grapevine leafroll-associated virus 3 by the vine mealybug (*Planococcus ficus*). *Phytopathology* **98**, 1093-1098 (2008).

18. Saporta, R., San Pedro, T. & Gisbert, C. Attempts at grapevine (*Vitis vinifera* L.) breeding through genetic transformation: The main limiting factors. *Vitis* **55**, 173-186 (2016).

19. Laimer, M. *et al.* Resistance to viruses, phytoplasmas and their vectors in the grapevine in Europe: A review. *J. Plant Pathol.* **91**, 7-23 (2009).

20. Shekhawat, U. K. S., Ganapathi, T. R. & Hadapad, A. B. Transgenic banana plants expressing small interfering RNAs targeted against viral replication initiation gene display high-level resistance to banana bunchy top virus infection. *J. Gen. Virol.* **93**, 1804-1813 (2012).

21. Younis, A., Siddique, M. I., Kim, C. K. & Lim, K. B. RNA interference (RNAi) induced gene silencing: A promising approach of hi-tech plant breeding. *Int. J. Biol. Sci.* **10**, 1150-1158 (2014).

22. Zhao, Y., Yang, X., Zhou, G. & Zhang, T. Engineering plant virus resistance: from RNA silencing to genome editing strategies. *Plant Biotechnol. J.* **18**, 328-336 (2020).

23. Levy, M., Edelbaum, O. & Sela, I. Tobacco mosaic virus regulates the expression of its own resistance gene N. *Plant Physiol.* **135**, 2392-2397 (2004).

24. Jones, J. D. & Dangl, J. L. The plant immune system. *Nature* **444**, 323-329 (2006).

25. Wiedenheft, B., Sternberg, S. H. & Doudna, J. A. RNA-guided genetic silencing systems in bacteria and archaea. *Nature* **482**, 331-338 (2012).

26. Sorek, R., Lawrence, C. M. & Wiedenheft, B. CRISPR-mediated adaptive immune systems in bacteria and archaea. *Annu. Rev. Biochem.* **82**, 237-266 (2013).

27. Cong, L. *et al.* Multiplex genome engineering using CRISPR/Cas systems. *Science* **339**, 819-823 (2013).

28. Ali, Z. *et al.* CRISPR/Cas9-mediated viral interference in plants. *Genome Biol.* **16**, 238 (2015).

29. Baltes, N. J. *et al.* Conferring resistance to geminiviruses with the CRISPR-Cas prokaryotic immune system. *Nat. Plants* **1**, 15145 (2015).

30. Ji, X., Zhang, H., Zhang, Y., Wang, Y. & Gao, C. Establishing a CRISPR-Cas-like immune system conferring DNA virus resistance in plants. *Nat. Plants* **1**, 15144 (2015).

31. Zhang, T. *et al.* Establishing RNA virus resistance in plants by harnessing CRISPR immune system. *Plant Biotechnol. J.* **16**, 1415-1423 (2018).

32. Aman, R. *et al.* RNA virus interference via CRISPR/Cas13a system in plants. *Genome Biol.* **19**, 1 (2018).

33. Pyott, D. E., Sheehan, E. & Molnar, A. Engineering of CRISPR/Cas9-mediated potyvirus resistance in transgene-free *Arabidopsis* plants. *Mol. Plant Pathol.* **17**, 1276-1288 (2016).

34. Chandrasekaran, J. *et al.* Development of broad virus resistance in non-transgenic cucumber using CRISPR/Cas9 technology. *Mol. Plant Pathol.* **17**, 1140-1153 (2016).

35. Mehta, D. *et al.* Linking CRISPR-Cas9 interference in cassava to the evolution of editing-resistant geminiviruses. *Genome Biol.* **20**, 80 (2019).

36. Gauffier, C. *et al.* A TILLING approach to generate broad-spectrum resistance to potyviruses in tomato is hampered by eIF4E gene redundancy. *Plant J.* **85**, 717-729 (2016).

37. Abudayyeh, O. O. *et al.* C2c2 is a single-component programmable RNA-guided RNA-targeting CRISPR effector. *Science* **353**, aaf5573 (2016).

38. Sampson, T. R., Saroj, S. D., Llewellyn, A. C., Tzeng, Y. L. & Weiss, D. S. A CRISPR/Cas system mediates bacterial innate immune evasion and virulence. *Nature* **497**, 254-257 (2013).

39. Price, A. A., Grakoui, A. & Weiss, D. S. Harnessing the prokaryotic adaptive immune system as a eukaryotic antiviral defense. *Trends Microbiol.* **24**, 294-306 (2016).

40. Ashraf, M. U. *et al.* CRISPR-Cas13a mediated targeting of hepatitis C virus internal-ribosomal entry site (IRES) as an effective antiviral strategy. *Biomed. Pharmacother.* **136**, 111239 (2021).

41. Zhang, T. *et al.* Establishing CRISPR/Cas13a immune system conferring RNA virus resistance in both dicot and monocot plants. *Plant Biotechnol. J.* **17**, 1185-1187 (2019).

42. Dolja, V. V., Kreuze, J. F. & Valkonen, J. P. Comparative and functional genomics of closteroviruses. *Virus Res.* **117**, 38-51 (2006).

43. Pang, X. *et al.* Involvement of calcium signalling in dormancy release of grape buds. *J. Exp. Bot.* **58**, 3249-3262 (2007).

44. Naidu, R., Rowhani, A., Fuchs, M., Golino, D. & Martelli, G. P. Grapevine leafroll: A complex viral disease affecting a high-value fruit crop. *Plant Dis.* **98**, 1172-1185 (2014).

45. Kurth, E. G. *et al.* Virus-derived gene expression and RNA interference vector for grapevine. *J. Virol.* **86**, 6002-6009 (2012).

46. Visser, M., Stephan, D., Jaynes, J. M. & Burger, J. T. A transient expression assay for the *in planta* efficacy screening of an antimicrobial peptide against grapevine bacterial pathogens. *Lett. Appl. Microbiol.* **54**, 543-551 (2012).

47. Jelly, N. S., Valat, L., Walter, B. & Maillot, P. Transient expression assays in grapevine: A step towards genetic improvement. *Plant Biotechnol. J.* **12**, 1231-1245 (2014).

48. Santos-Rosa, M., Poutaraud, A., Merdinoglu, D. & Mestre, P. Development of a transient expression system in grapevine via agro-infiltration. *Plant Cell Rep.* **27**, 1053-1063 (2008).

49. Xu, W., Yu, Y., Ding, J., Hua, Z. & Wang, Y. Characterization of a novel stilbene synthase promoter involved in pathogen- and stress-inducible expression from Chinese wild *Vitis pseudoreticulata*. *Planta* **231**, 475-487 (2010).

50. Le Henanff, G. *et al.* Characterization of *Vitis vinifera* NPR1 homologs involved in the regulation of pathogenesis-related gene expression. *BMC Plant Biol.* **9**, 54 (2009).

51. Zottini, M. *et al.* Agroinfiltration of grapevine leaves for fast transient assays ofgene expression and for long-term production of stable transformed cells. *Plant Cell Rep.* **27**, 845-853 (2008).

52. Zhan, X. *et al.* Generation of virus-resistant potato plants by RNA genome targeting. *Plant Biotechnol. J.* **17**, 1814-1822 (2019).

53. Acharya, S. *et al.* *Francisella novicida* Cas9 interrogates genomic DNA with very high specificity and can be used for mammalian genome editing. *Proc. Natl. Acad. Sci. U S A* **116**, 20959-20968 (2019).

54. Abbott, T. R. *et al.* Development of CRISPR as an antiviral strategy to combat SARS-CoV-2 and influenza. *Cell* **181**, 865-876 (2020).

55. Freije, C. A. *et al.* Programmable inhibition and detection of RNA viruses using Cas13. *Mol. Cell* **76**, 826-837 (2019).

56. Yan, W. X. *et al.* Cas13d is a compact RNA-targeting type VI CRISPR effector positively modulated by a WYL-domain-containing accessory protein. *Mol. Cell* **70**, 327-339 (2018).

57. Abudayyeh, O. O. *et al.* RNA targeting with CRISPR-Cas13. *Nature* **550**, 280-284 (2017).

58. Cox, D. B. T. *et al.* RNA editing with CRISPR-Cas13. *Science* **358**, 1019-1027 (2017).

59. Mahas, A., Aman, R. & Mahfouz, M. CRISPR-Cas13d mediates robust RNA virus interference in plants. *Genome Biol.* **20**, 263 (2019).

60. Bester, R., Pepler, P. T., Burger, J. T. & Maree, H. J. Relative quantitation goes viral: An RT-qPCR assay for a grapevine virus. *J. Virol. Methods* **210**, 67-75 (2014).

61. Cui, Z. H. *et al.* Responses of *in vitro*-grown plantlets (*Vitis vinifera*) to grapevine leafroll-associated virus-3 and PEG-induced drought stress. *Front. Physiol.* **7**, 203 (2016).

62. Cui, Z. H., Bi, W. L., Pan, C., Xu, Y. & Wang, Q. C. Abiotic stress improves *in vitro* biological indexing of grapevine leafroll-associated virus-3 in red grapevine cultivars. *Aust. J. Grape Wine Res.* **21**, 490-495 (2015).

63. Cui, Z. H. *et al.* Drought stress enhances up-regulation of anthocyanin biosynthesis in grapevine leafroll-associated virus 3-infected *in vitro* grapevine (*Vitis vinifera*) leaves. *Plant Dis.* **101**, 1606-1615 (2017).

64. Wise, A. A., Liu, Z. & Binns, A. N. Three methods for the introduction of foreign DNA into Agrobacterium. in Agrobacterium protocols 43–54 (Springer, 2006).

65. Baltes, N. J., Gil-Humanes, J., Cermak, T., Atkins, P. A. & Voytas, D. F. DNA replicons for plant genome engineering. *Plant Cell* **26**, 151-163 (2014).

66. Osman, F. & Rowhani, A. Application of a spotting sample preparation technique for the detection of pathogens in woody plants by RT-PCR and real-time PCR (TaqMan). *J. Virol. Methods* **133**, 130-136 (2006).

67. Chen, T. *et al.* Importin-αs are required for the nuclear localization and function of the *Plasmopara viticola* effector PvAVH53. *Hortic. Res.* **8**, 46 (2021).
