# Supplementary material for: Engineering CRISPR immune systems conferring GLRaV-3 resistance in grapevine
Source: Hortic Res. 2022 Jan 28;9:uhab023. doi: 10.1093/hr/uhab023 (PMC8796251; doi:10.1093/hr/uhab023)
Supplement: Web_Material_uhab023 [file web_material_uhab023.zip › Supplementary Table S1. Primers used for vector construction, detection and RT-qPCR.docx]

**Supplementary Table S1. Primers used for vector construction, detection and RT-qPCR**

| **Primer** | **Sequence (5'-3')** | **Purpose** | **Reference** |
| --- | --- | --- | --- |
| 01-GUS-F | TAATAACTGAggatccGCTACTAACTTCAGCCTGCT | Vector construction | This study |
| 01-GUS-R | CGATCAATCAggatccTTACTTGTACAGCTCGTCCATGC |  |  |
| 11-GUS-F | CTATGCCTAAggatccGCTACTAACTTCAGCCTGCT | Vector construction | This study |
| 11-GUS-R | CGATCAATCAggatccTTACTTGTACAGCTCGTCCATGC |  |  |
| sgRNA F: | TCAAAAGTCCCACATCGCTT | RT-PCR for sgRNA | This study |
| sgRNA R: | CGCTACGGACTAGCCTTATT |  |  |
| GLRaV-3-CP-F: | ATGCGGCACAAGGAAAGTTTAG | RT-PCR for GLRaV-3 CP RNA | This study |
| GLRaV-3-CP-R: | GCTGTAGCCAAAGCTGCTGT |  |  |
| GLRaV-3 F： | CGCTAGGGCTGTGGAAGTATT | Detection for GLRaV-3 RNA | (Osman and Rowhani, 2006) |
| GLRaV-3 R： | GTTGTCCCGGGTACCAGATAT |  |  |
| qVvActin-F | GTGACGGAGAATTAGGGTTCGA | RT-qPCR for Actin of *Vitis vinifera* L. | (Osman and Rowhani, 2006) |
| qVvActin-R | CTGCCTTCCTTGGATGTGGTA |  |  |
| qGLRaV-3-F: | GGGRACGGARAAGTGTTACC | RT-qPCR for GLRaV-3 RNA | (Bester *et al.*, 2014) |
| qGLRaV-3-R: | TCCAAYTGGGTCATRCACAA |  |  |
| qFnCas9-F: | GATAAGGACACACAGCAGGCGAT | RT-qPCR for FnCas9 expression | (Zhang *et al.*, 2018) |
| qFnCas9-R: | CTGCTCTGTCGCGAGCTTCAG |  |  |
| qLshCas13a-F: | CAGGAGAACGAGGAAGAGATCGA | RT-qPCR for LshCas13a expression | (Zhang *et al.*, 2019) |
| qLshCas13a-R: | CAGTCTCGTTCTCGATGATCTTCTC |  |  |
