# Supplementary material for: Engineering CRISPR immune systems conferring GLRaV-3 resistance in grapevine
Source: Hortic Res. 2022 Jan 28;9:uhab023. doi: 10.1093/hr/uhab023 (PMC8796251; doi:10.1093/hr/uhab023)
Supplement: Web_Material_uhab023 [file web_material_uhab023.zip › Supplementary Table S2. Sequence of sgRNA used in this study.docx]

**Supplementary Table S2. Sequence of sgRNA used in this study**

| **Targets in GLRaV-3** | **sgRNA-ID** | **Target sequence** | **Oligonucleotide sequence** | |
| --- | --- | --- | --- | --- |
| p5 | 1A | AACGGAATTGATCAACACC | F： | gattGGTGTTGATCAATTCCGTT |
|  |  |  | R： | cgagAACGGAATTGATCAACACC |
| Hsp70h | 1B | CAGCCCTCTATTCCTTAGC | F： | gattGCTAAGGAATAGAGGGCTG |
|  |  |  | R： | cgagCAGCCCTCTATTCCTTAGC |
| Hsp90h | 1C | CTGCTAGTACGAAGACGAC | F： | gattGTCGTCTTCGTACTAGCAG |
|  |  |  | R： | cgagCTGCTAGTACGAAGACGAC |
| CP | 1D | CTTAAAATACGTTAAGGAC | F： | gattGTCCTTAACGTATTTTAAG |
|  |  |  | R： | cgagCTTAAAATACGTTAAGGAC |
| CPm | 1E | GAGGTTACAGCAAAGCTCC | F： | gattGGAGCTTTGCTGTAACCTC |
|  |  |  | R： | cgagGAGGTTACAGCAAAGCTCC |
