# Supplementary material for: Engineering CRISPR immune systems conferring GLRaV-3 resistance in grapevine
Source: Hortic Res. 2022 Jan 28;9:uhab023. doi: 10.1093/hr/uhab023 (PMC8796251; doi:10.1093/hr/uhab023)
Supplement: Web_Material_uhab023 [file web_material_uhab023.zip › Supplementary Table S3. Sequence of crRNA used in this study.docx]

**Supplementary Table S3. Sequence of crRNA used in this study**

| **Targets in GLRaV-3** | **crRNA-ID** | **Target sequence** | **Oligonucleotide sequence** | |
| --- | --- | --- | --- | --- |
| p5 | 2A | ATTACGTTTGTCGTCCCGAGGTTACAGC | F： | aaacGCTGTAACCTCGGGACGACAAACGTAAT |
|  |  |  | R： | aaaaATTACGTTTGTCGTCCCGAGGTTACAGC |
| Hsp70h | 2B | ACTGTGATATTTTGGCAGGTAATAGCGG | F： | aaacCCGCTATTACCTGCCAAAATATCACAGT |
|  |  |  | R： | aaaaACTGTGATATTTTGGCAGGTAATAGCGG |
| Hsp90h | 2C | CGGCCTTAATAATTATGCTGATTTACTA | F： | aaacTAGTAAATCAGCATAATTATTAAGGCCG |
|  |  |  | R： | aaaaCGGCCTTAATAATTATGCTGATTTACTA |
| CP | 2D | ACGTTAAGGACGGGACGCAGGCGGAGTT | F： | aaacAACTCCGCCTGCGTCCCGTCCTTAACGT |
|  |  |  | R： | aaaaACGTTAAGGACGGGACGCAGGCGGAGTT |
| CPm | 2E | AGCGCCAAGTCCAGTAGCACCAGCGCCT | F： | aaacAGGCGCTGGTGCTACTGGACTTGGCGCT |
|  |  |  | R： | aaaaAGCGCCAAGTCCAGTAGCACCAGCGCCT |
